# Supplementary material for: Long-term mammal herbivory on arthropod assemblages at Kruger National Park, South Africa
Source: PLoS One. 2023 Jun 2;18(6):e0286396. doi: 10.1371/journal.pone.0286396 (PMC10237461; doi:10.1371/journal.pone.0286396)
Supplement: S1 Table — Species classified to the order level were classified as “Others”, but were distinguished by the unique morphological characteristics. (DOCX) [file pone.0286396.s001.docx]

S1 Table: Numbers of morphospecies and their abundances for arthropods sampled at the ungrazed, moderately and heavily grazed exclosures of Kruger National Park. Species classified to the order level were classified as “Others” but were distinguished by the unique morphological characteristics.

| Order | Family/ **Subfamily** | No. of Morphospecies | Abundance | Singletons | % Accum. Singletons |
| --- | --- | --- | --- | --- | --- |
| Araneae | Agelenidae | 1 | 3 |  | 0 |
|  | Antrodraetidae | 2 | 3 | 2 | 1.8 |
|  | Buthidae | 1 | 4 | 1 | 2.7 |
|  | Lycosidae | 2 | 3 | 2 | 4.5 |
|  | Salticidae | 2 | 4 |  | 4.5 |
|  | Sicariidae | 1 | 1 | 1 | 5.4 |
|  | Sparassidae | 1 | 1 | 1 | 6.3 |
|  | Thomisidae | 1 | 1 | 1 | 7.2 |
|  | Others(s) | 6 | 12 | 4 | 10.8 |
| Blattodea | Blaberidae | 4 | 11 | 2 | 12.6 |
|  | Hodotermitidae | 2 | 2 | 2 | 14.4 |
|  | Pseudophyllodromidae | 1 | 2 | 1 | 15.3 |
|  | Termitidae | 1 | 8 |  | 15.3 |
| Coleoptera | Carabidae | 7 | 51 | 2 | 17.1 |
|  | Curculionidae | 3 | 9 | 1 | 18 |
|  | Gyrinidae | 2 | 385 |  | 18 |
|  | Hydrophilidae | 2 | 37 |  | 18 |
|  | Staphylinidae | 1 | 1 | 1 | 18.9 |
|  | Tenebrionidae | 10 | 259 | 5 | 23.4 |
|  | Other(s) | 7 | 56 |  | 23.4 |
| Diptera | Bombylidae | 2 | 4 | 1 | 24.3 |
|  | Calliphoridae | 2 | 11 | 1 | 25.2 |
|  | Musadae | 3 | 20 |  | 25.2 |
|  | Tabanidae | 2 | 2 | 2 | 27 |
| Hemiptera | Reduviidae | 6 | 10 | 4 | 30.6 |
|  | Other(s) | 1 | 2 | 1 | 31.5 |
| Hymenoptera | Formicidae/ **Formicinae** | 7 | 389 | 3 | 34.2 |
|  | Formicidae/ **Myrmicinae** | 13 | 1601 | 5 | 38.7 |
|  | Formicidae/ **Ponerinae** | 6 | 581 | 1 | 39.6 |
|  | Formicidae/ **Pseudomyrmecinae** | 1 | 16 |  | 39.6 |
| Lepidoptera | Nymphalidae | 3 | 12 |  | 39.6 |
|  | Butterfly1 | 1 | 2 |  | 39.6 |
| Orthoptera | Acrididae1 | 4 | 9 | 2 | 41.4 |
|  | Anostostomatidae1 | 1 | 5 |  | 41.4 |
|  | Gryllacrididae1 | 1 | 1 | 1 | 42.3 |
|  | Gryllidae1 | 1 | 8 |  | 42.3 |
| **Total** |  | **111** | **3526** | **47** | **42.3** |
