# Supplementary material for: Long-term mammal herbivory on arthropod assemblages at Kruger National Park, South Africa
Source: PLoS One. 2023 Jun 2;18(6):e0286396. doi: 10.1371/journal.pone.0286396 (PMC10237461; doi:10.1371/journal.pone.0286396)
Supplement: S2 Table — (DOCX) [file pone.0286396.s002.docx]

**S2 Table**: SIMPER analysis showing similarity of multi-taxon sampled at the ungrazed, moderately and heavily grazed exclosures of Kruger National Park.

| Exclosure name | Average similarity | Morphospecies | Av.Abund | Av.Sim | Sim/SD | Contrib% | Cum.% |
| --- | --- | --- | --- | --- | --- | --- | --- |
| Ungrazed | 17.50 | *Pheidole sp.* 01 | 1,85 | 6,42 | 0,51 | 36,67 | 36,67 |
|  |  | Gyrinidae 1 | 1,14 | 3,89 | 0,48 | 22,24 | 58,91 |
|  |  | Tenebrionidae 1 | 0,47 | 1,44 | 0,3 | 8,24 | 67,15 |
|  |  | *Bothroponera sp*. 01 | 0,67 | 1,08 | 0,2 | 6,16 | 73,31 |
|  |  | *Ocymyrmex sp.* 01 | 0,54 | 0,97 | 0,24 | 5,54 | 78,85 |
|  |  | *Plagiolepis sp.* 01 | 0,49 | 0,57 | 0,15 | 3,25 | 82,1 |
|  |  | *Anoplolepis custodiens* | 0,48 | 0,56 | 0,18 | 3,23 | 85,32 |
|  |  | *Crematogaster castanea* | 0,39 | 0,37 | 0,12 | 2,13 | 87,45 |
|  |  | *Lepisiota sp.* 01 | 0,24 | 0,31 | 0,17 | 1,75 | 89,2 |
|  |  | *Hagensia havilandi* | 0,29 | 0,28 | 0,14 | 1,61 | 90,81 |
| Moderately grazed | 16.31 | *Bothroponera sp*. 01 | 1,28 | 6,21 | 0,53 | 38,09 | 38,09 |
|  |  | Gyrinidae 1 | 0,87 | 4,45 | 0,46 | 27,28 | 65,37 |
|  |  | *Pheidole sp.* 01 | 0,68 | 1,58 | 0,22 | 9,69 | 75,06 |
|  |  | Tenebrionidae 1 | 0,41 | 1,11 | 0,27 | 6,82 | 81,88 |
|  |  | *Lepisiota sp.* 01 | 0,32 | 0,65 | 0,21 | 3,99 | 85,87 |
|  |  | *Monomorium junodi* | 0,35 | 0,49 | 0,18 | 3 | 88,87 |
|  |  | *Ocymyrmex sp*. 01 | 0,35 | 0,3 | 0,1 | 1,85 | 90,72 |
| Heavily grazed | 12.79 | *Pheidole sp.* 01 | 1,04 | 3,86 | 0,42 | 30,19 | 30,19 |
|  |  | Tenebrionidae 1 | 0,47 | 1,25 | 0,28 | 9,76 | 39,96 |
|  |  | *Tetramorium sp*. 01 | 0,51 | 1,2 | 0,29 | 9,35 | 49,3 |
|  |  | *Monomorium junodi* | 0,42 | 0,8 | 0,24 | 6,23 | 55,53 |
|  |  | *Bothroponera sp*. 01 | 0,36 | 0,75 | 0,18 | 5,85 | 61,38 |
|  |  | Gyrinidae 1 | 0,41 | 0,69 | 0,22 | 5,38 | 66,77 |
|  |  | Beetle 2 | 0,27 | 0,55 | 0,22 | 4,28 | 71,05 |
|  |  | *Ocymyrmex sp*. 01 | 0,24 | 0,4 | 0,17 | 3,12 | 74,17 |
|  |  | *Monomorium sp*. 01 | 0,32 | 0,38 | 0,12 | 3,01 | 77,18 |
|  |  | Tenebrionidae 2 | 0,24 | 0,35 | 0,19 | 2,77 | 79,95 |
|  |  | *Lepisiota sp*. 01 | 0,21 | 0,34 | 0,15 | 2,64 | 82,59 |
|  |  | *Hagensia havilandi* | 0,24 | 0,26 | 0,14 | 2,05 | 84,64 |
|  |  | Hydrophilidae 1 | 0,22 | 0,26 | 0,14 | 2,02 | 86,66 |
|  |  | *Anoplolepis custodiens* | 0,22 | 0,22 | 0,12 | 1,75 | 88,41 |
|  |  | *Polyrhachis schistacea* | 0,22 | 0,22 | 0,12 | 1,74 | 90,15 |
